# Deficiency of eIF4B Increases Mouse Mortality and Impairs Antiviral Immunity

**DOI:** 10.3389/fimmu.2021.723885

**Published:** 2021-09-10

**Authors:** Biao Chen, Yuhai Chen, Kul Raj Rai, Xuefei Wang, Shasha Liu, Yingying Li, Meng Xiao, Yun Ma, Guoqing Wang, Guijie Guo, Shile Huang, Ji-Long Chen

**Affiliations:** ^1^CAS Key Laboratory of Pathogenic Microbiology and Immunology, Institute of Microbiology, Chinese Academy of Sciences (CAS), Beijing, China; ^2^College of Life Sciences, University of Chinese Academy of Sciences, Beijing, China; ^3^College of Animal Sciences, Fujian Agriculture and Forestry University, Fuzhou, China; ^4^Department of Biochemistry and Molecular Biology, Louisiana State University Health Sciences Center, Shreveport, LA, United States

**Keywords:** eIF4B, mouse mortality, viral infection, inflammation, antiviral immunity

## Abstract

Eukaryotic translation initiation factor 4B (eIF4B) plays an important role in mRNA translation initiation, cell survival and proliferation *in vitro*. However, its function *in vivo* is poorly understood. Here, we identified that eIF4B knockout (KO) in mice led to embryonic lethality, and the embryos displayed severe liver damage. Conditional KO (CKO) of eIF4B in adulthood profoundly increased the mortality of mice, characterized by severe pathological changes in several organs and reduced number of peripheral blood lymphocytes. Strikingly, eIF4B CKO mice were highly susceptible to viral infection with severe pulmonary inflammation. Selective deletion of eIF4B in lung epithelium also markedly promoted replication of influenza A virus (IAV) in the lung of infected animals. Furthermore, we observed that eIF4B deficiency significantly enhanced the expression of several important inflammation-associated factors and chemokines, including serum amyloid A1 (Saa1), Marco, Cxcr1, Ccl6, Ccl8, Ccl20, Cxcl2, Cxcl17 that are implicated in recruitment and activation of neutrophiles and macrophages. Moreover, the eIF4B-deficient mice exhibited impaired natural killer (NK) cell-mediated cytotoxicity during the IAV infection. Collectively, the results reveal that eIF4B is essential for mouse survival and host antiviral responses, and establish previously uncharacterized roles for eIF4B in regulating normal animal development and antiviral immunity *in vivo*.

## Introduction

Protein synthesis is a fundamental and intricate biological process in eukaryotic cells, which is crucial for cell survival, growth, proliferation, migration and differentiation (1, 2). Translation is generally divided into four steps: initiation, elongation, termination, and ribosome recycling (3). Of note, translation initiation is a major rate-limiting step of protein synthesis and is often the effective target for complex regulatory mechanisms (3, 4). Translation of messenger RNA (mRNA) is initiated by the assembly of eukaryotic translation initiation factor (eIF) 4F (eIF4F) complex (5, 6), which contains three key initiation factors: eIF4E (recognizing and binding the mRNA 5’ cap structures) (7, 8), eIF4A (functioning as the ATP-dependent RNA helicase) (9, 10), and eIF4G (acting as a scaffold) (3, 11). Mounting evidence indicates that eIF4B is also important for translation initiation. It has been shown that eIF4B not only participates in the recruitment of ribosome in the process of eukaryotic translation initiation, but also plays a key role in stimulating the RNA helicase activity of eIF4A (3, 5, 12–14), which results in more effective unwinding of the complex mRNA secondary structure in the 5’ untranslated region and translation of these mRNAs (15). Thus, eIF4B is also a crucial protein in regulating translation initiation.

It has been demonstrated that eIF4B has multiple phosphorylation sites (16–18), which can be mediated by various protein kinases (14, 19–24). For example, in response to serum stimulation, p70S6 kinases (S6K1/S6K2) can phosphorylate eIF4B at Ser422, which is sensitive to inhibitors of PI3K and mTOR (21). Besides, Akt can also phosphorylate eIF4B at Ser422 independently of the activity of either S6K or mitogen-activated protein kinase (MAPK) (22). Furthermore, eIF4B can be phosphorylated at Ser406 by maternal and embryonic leucine zipper kinase (MELK), a serine/threonine (Ser/Thr) kinase belonging to the AMP-activated protein kinase (AMPK) family (23). In addition, our previous studies have shown that the Pim kinases (Pim-1 and Pim-2) can directly phosphorylate eIF4B at Ser406 and Ser422 in Abl-transformed cells, and downregulation of eIF4B impairs the transforming efficiency medicated by Bcr-Abl or v-Abl (24). Recently, Ser504 of eIF4B has been identified as a new phosphorylation site, which is regulated by casein kinase in neurons, affects the localization of eIF4B at synapses, and controls the synaptic plasticity by modulating translation (19).

Moreover, noncoding RNAs can directly interact with eIF4B and regulate its protein expression and phosphorylation as well. It has been described that regulatory brain cytoplasmic (BC) RNAs bind eIF4B, repressing the translation initiation in neurons; upon neuronal stimulation, protein phosphatase 2A (PP2A) can dephosphorylate eIF4B at Ser406, reducing the binding affinity of BC RNAs to eIF4B and thus increasing translation (20). Additionally, lncRNA-GMAN directly combines with eIF4B, which interferes with the interaction of eIF4B and PP2A B subunit, thereby preventing the dephosphorylation of eIF4B at Ser422 and subsequently increasing the mRNA translation of anti-apoptosis proteins in hepatocellular carcinoma cells (25). Together, these findings suggest that eIF4B can be regulated by diverse signaling molecules related to cell survival, proliferation, and differentiation.

It is known that eIF4B acts as a common substrate of several crucial proto-oncogenic signaling pathways, such as the RAS-MAPK and PI3K/mTOR pathways (14, 21, 22, 26). Phosphorylated eIF4B is involved in regulating translation initiation of some proliferative (Cdc25c and c-Myc) and anti-apoptotic (Bcl-2 and XIAP) mRNAs with inhibitory secondary structures in the 5’ UTR and subsequently promotes cell proliferation and survival (27–29). Therefore, dysregulation of eIF4B protein expression or its phosphorylation is associated with the development and progression of numerous human diseases including Alzheimer’s disease (30), diffuse large B-cell lymphoma (DLBCL) (31, 32), T-cell lymphoblastic leukemia (33), acute myeloid leukemia (34), Abl-positive CML (24), breast cancer (35, 36), and hepatocellular carcinoma (25, 37). It has been noticed that overexpression of eIF4B in DLBCL patients enhances the expression of several key proteins (DAXX, BCL2 and ERCC5), while reduced expression of eIF4B decreases the synthesis of those proteins and tumor cell survival, so the expression levels of eIF4B, ERCC5 and DAXX are directly correlated with the survival rate of patients (31). In addition, inhibition of matrix metallopeptidase 13 (MMP13)-mediated phosphorylation of eIF4B (Ser422) reduces the protein synthesis of β-site amyloid precursor protein cleaving enzyme 1 (BACE1) and amyloid-β accumulation, which contributes to the improved learning and memory in the animal model of Alzheimer’s disease (30).

Interestingly, dysregulation of eIF4B expression or its phosphorylation also affects the interaction between virus and host (28, 38–40). It has been shown that influenza A virus (IAV) infection induces the degradation of eIF4B protein through the lysosomal pathway in A549 cells and in multiple organs of mice, and downregulation of eIF4B remarkably enhances the IAV replication (40). Also, eIF4B plays an important role in antiviral immunity during influenza virus infection by regulating the expression of interferon (IFN)-induced transmembrane protein 3 (IFITM3), a critical IFN-stimulated gene (ISG) involved in innate immune response (40). Besides, S6K-mediated phosphorylation of eIF4B (Ser422) has been implicated in IFN-induced protein expression of ISG15 and CXCL-10 (38). Some viruses also take advantage of the activity of eIF4B to promote the translation of viral proteins required for viral replication. For example, open reading frame 45 (ORF45) of Kaposi sarcoma-associated herpesvirus (KSHV) induces the activation of eIF4B, which, in turn, increases KSHV lytic gene expression and promotes the production of progeny viruses (28).

Although the function of eIF4B has been extensively studied at the cellular and molecular levels *in vitro*, its physiological role *in vivo* is largely unknown. Here, we show that germ-line knockout (KO) of eIF4B in mice results in severe defect in fetal liver development, leading to embryonic lethality. Conditional KO of eIF4B in adulthood increases the mortality of adult mice and causes the atrophy of immune organs including spleen and thymus. Furthermore, eIF4B-deficient mice are more susceptible to viral infection; selective deletion of eIF4B in lung epithelium significantly enhances viral replication in the lung. Our findings demonstrate that eIF4B is essential for mouse survival and antiviral immunity.

## Materials and Methods

### Ethics Statement

All animal experiments in this study were approved by the Research Ethics Committee of Institute of Microbiology, Chinese Academy of Sciences (Permit Number: SQIMCAS2019033). All mouse experimental procedures were conducted in accordance with the Regulations for the Administration of Affairs Concerning Experimental Animals approved by the State Council of People’s Republic of China.

### Generation of eIF4B Knockout Mouse Models

**Figure 1:**
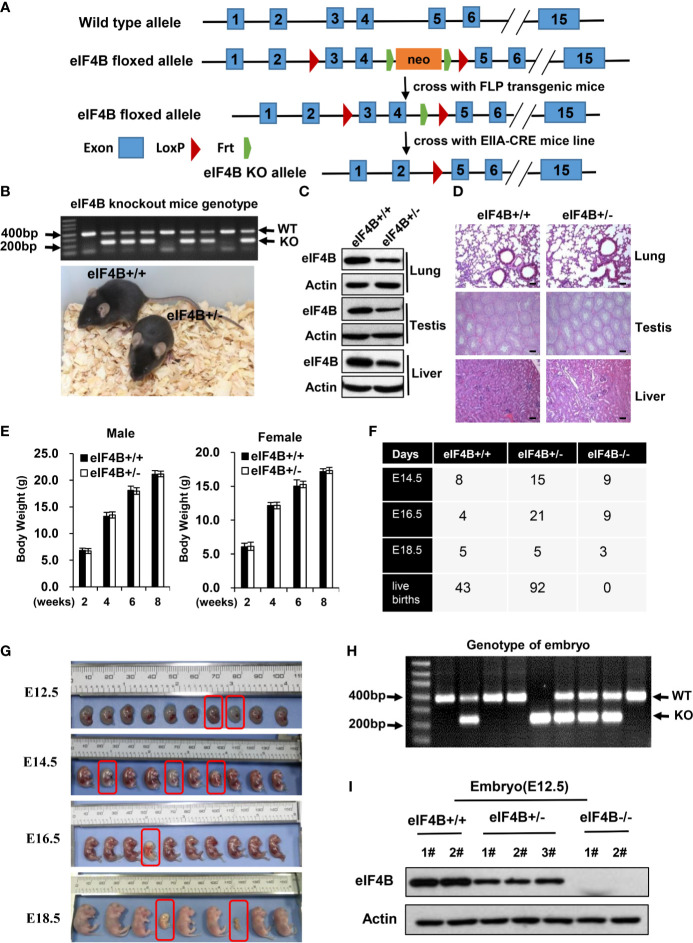
Knockout of eIF4B leads to mouse embryonic lethality. **(A)** Schematic representation of the targeted eIF4B locus. Exons 3 and 4 are targeted and flanked by two LoxP sits upon Frt-flanked neomycin cassette. **(B)** The genotypes and representative images of eIF4B^+/-^ mice and their eIF4B^+/+^ littermates. **(C)** Protein levels of eIF4B in the indicated tissues of eIF4B^+/-^ mice and their eIF4B^+/+^ littermates were detected by Western blotting. **(D)** Representative micrographs of the indicated organs stained with hematoxylin and eosin (HE) from eIF4B^+/-^ mice and their eIF4B^+/+^ littermates. Scale bars, 100 µm. **(E)** Comparing the average weights of eIF4B^+/-^ mice and their eIF4B^+/+^ littermates within two months of age (n = 4-7 per genotype for each group). Data are represented as mean ± SD. **(F)** Statistics of offspring derived from mating eIF4B^+/-^ female and male mice. **(G)** Gross images of E12.5, E14.5, E16.5, and E18.5 embryos derived from mating eIF4B^+/-^ female and male mice. **(H)** Representative genotypes of embryos by PCR. **(I)** Protein levels of eIF4B in embryos were detected by Western blotting.

eIF4B^f/f^ mice were generated by targeting the mouse *EIF4B* locus with an rDNA replacement vector, two loxP sites flanking exons 3/4 and the Frt-flanked neomycin cassette were inserted in the downstream intron 2 (**Figure 1A**). Then the generated eIF4B^f/f^ mice were initially crossed to a transgenic line expresses Flp recombinase to eliminate Frt-flanked neomycin cassette. eIF4B conventional knockout mice (eIF4B^+/^ **^-^**) were generated by crossing eIF4B^f/f^ mice with EIIA-cre transgenic mice. eIF4B conditional knockout mice eIF4B^f/f^ UBC-CreER^T2^ were generated by crossing eIF4B^f/f^ mice with UBC-CreER^T2^ transgenic mice. eIF4B^f/f^ mice were mated with SPC-rtTA/Teto-Cre mice to obtain eIF4B^f/f^ SPC-rtTA/Teto-Cre mice in which *EIF4B* gene is conditionally inactivated in lung epithelium by doxycycline treatment. eIF4B^+/+^ and eIF4B^+/-^ mice were genotyped by PCR using genomic DNA isolated from tail tips with primers: eIF4B-P1 (5’- TCTGTGTAG CCCTGGCTATGCTAA -3’), eIF4B-P2 (5’- ATCAGCGCTGTACGCTTACCACG -3’), eIF4B-P3 (5’- TTCCATTGCAATCACTGTACCTG -3’). All mice were housed and bred under specific pathogen–free conditions. eIF4B^f/f^ mice were constructed in Shanghai Model Organisms Center (Shanghai, China).

### Conditional Knockout of eIF4B in Mice

To conditionally knockout eIF4B in adult mice, tamoxifen [20 mg/ml, dissolved in a mixture of 98% corn oil and 2% ethanol (41)] was delivered into 10-12 weeks-old eIF4B^f/f^ UBC-CreER^T2^ mice by intraperitoneal injection (150 µg/g body weight, once per day for 5 consecutive days). To obtain eIF4B-deficient mice for virus infection, tamoxifen was delivered into 6 weeks-old eIF4B^f/f^ UBC-CreER^T2^ mice by intraperitoneal injection (100 µg/g body weight, once per day for 3 consecutive days). To obtain lung epithelium-specific deletion of eIF4B, eIF4B^f/f^ SPC-rtTA/Teto-Cre mice were treated with doxycycline (1 mg/ml) in drinking water. Doxycycline water was replaced three times per week (42).

### Viral Infection and Lung Virus Determination

Tamoxifen was delivered into 6 weeks-old eIF4B^f/f^ and eIF4B^f/f^ UBC-CreER^T2^ mice by intraperitoneal injection (100 µg/g body weight, once per day for 3 consecutive days). On day five after first Tamoxifen treatment, mice were inoculated intranasally with influenza virus (WSN), PRV at the indicated time post-infection as previously described (40, 43). For virus infection, mice were inoculated with 5×10^4^ PFU of virus. Lung viral load was determined at 72 h post-infection. Lungs of infected mice were homogenized in 1 ml of ice-cold PBS and frozen at -80°C for 14 h. Then, thawed samples were centrifuged at 12,000 ×*g* for 10 min, and the supernatants were titrated by plaque forming assay as described previously (40).

### RNA-Seq Analysis

Wild type and eIF4B CKO mice were infected with WSN (5×10^4^ PFU) or Mock for 48 h. Lungs of these mice were used for analysis by transcriptome RNA-sequencing. The RNA-seq data have been deposited on GEO public database under the accession number GSE166944.

### Isolation of Mouse Embryonic Fibroblasts

eIF4B^+/+^, eIF4B^+/-^ and eIF4B^-/-^ mouse embryonic fibroblasts (MEFs) were isolated from E12.5–E14.5 embryos as described (40). MEFs were maintained in Dulbecco’s modified Eagle’s medium (DMEM) containing 10% fetal calf serum (FCS) supplemented with penicillin and streptomycin.

### Histopathological Analysis

Mouse organs were fixed in 4% paraformaldehyde and embedded in paraffin. Then, 4-mm-thick sections were prepared and stained with hematoxylin and eosin (HE). The slides were visualized under an Olympus BH-2 microscope (Tokyo, Japan).

### Fetal Liver Cell Counting

Fetal livers were dissected from eIF4B^+/+^ and eIF4B^-/-^ embryos at E14.5, and collected in 1 ml PBS contained 2% FCS. To obtain signal-cell suspensions, the fetal livers were gently pipetted up and down using a 1-ml pipette and the suspensions were filtered through a 40-μm nylon cell strainer (BD Biosciences, San Jose, CA, USA). Then, the cell number was counted by a hemocytometer (44).

### Flow Cytometric Analysis of Lung Cells

Lung cell isolation and flow cytometric analysis were performed as described previously (45). Briefly, lung single-cell suspensions were obtained from minced lung tissue and subjected to digestion with collagenase/dispase (1 mg/ml; Roche) and DNase I (100 µg/ml; grade II; Roche), then treated with RBC lysis buffer to remove red blood cells. Cells were suspended in PBS with 1% BSA, blocked with 2.4G2 (anti-CD16/CD32, Multi Sciences, Hangzhou, China), and labeled with specific antibodies. Isolation of lung lymphocytes for detection of NK cells was performed as described (46). Antibodies used included: SiglecF, Ly6C, CD19, CD4, CD8 (eBioscience, San Diego, CA, USA), CD45, Ly6G, CD11c, CD11b, CD3e, NK1.1 (Multi Sciences), CD107a, IFN-γ (BD Biosciences). Flow cytometry was used to analyze specific cell populations in mouse lungs using the following gates: AMs as SiglecF^+^CD11c^+^, eosinophils as SiglecF^+^CD11c^-^, tissue monocytes as SiglecF^-^ Ly6G^-^CD11b^+^Ly6C^+^, and neutrophils as Ly6G^+^CD11b^+^.

### Western Blotting and Antibodies

Cell and tissue lysates were prepared, and Western blotting was performed as described previously (47). Briefly, samples were separated on SDS-polyacrylamide gel, transferred onto a nitrocellulose membrane, and probed with antibodies as indicated. In this study we used antibodies to eIF4B (SC-376062, Santa Cruz Biotechnology, Dallas, TX, USA), The polyclonal antibody against IAV NP was generated and used in our lab as previously described (47, 48).

### RNA Extraction, RT-PCR, and Quantitative PCR

Total RNA was extracted from cells or tissues using TRIzol reagent (Invitrogen, Carlsbad, CA, USA). cDNA was synthesized using 5 µg of total RNA and GoScript reverse transcriptase (Promega, Madison, WI, USA), followed by PCR using rTaq DNA polymerase and quantitative PCR using KAPA HRM FAST qPCR Master Mix (2X) Kits (KAPA BIOSYSTEMS, Indianapolis, IN, USA) with the specific primers. Actin was chosen as a reference gene for internal standardization.

### Statistical Analysis

Statistical significance was determined by Student’s *t*-test. All data represent the mean ± SD and p values < 0.05 was considered to be statistically significant.

## Results

### Knockout of eIF4B Leads to Mouse Embryonic Lethality

Our *in vitro* studies demonstrate that eIF4B plays an important role in tumorigenesis (24, 49). To explore the physiologic function of eIF4B *in vivo*, we generated an eIF4B knockout (KO) mouse model (eIF4B^f/f^ mice) (**Figure 1A**). Firstly, the generated eIF4B^f/f^ mice were crossed to an FLP transgenic mouse line to delete the neomycin cassette, and then crossed to germline EIIa-cre mice carrying a cre transgene that encodes a cre recombinase and can be induced in the early mouse embryo to generate eIF4B conventional knockout mice (**Figures 1A, B**). We observed that heterozygous (eIF4B^+/-^) mice showed approximately 50% reductions in eIF4B level in various tissues (**Figure 1C** and **Supplementary Figure 1A**). Both eIF4B^+/-^ and wild type (WT) mice exhibited similar body weight within two months of age and normal tissue architecture by hematoxylin and eosin (HE) staining (**Figures 1D, E** and **Supplementary Figure 1B**), indicating that the 50% reductions in eIF4B level may not affect normal organ development. However, no mice with homozygous deletion of eIF4B (eIF4B^-/-^) were born after the eIF4B^+/-^ female and male mice were crossbred each other (**Figure 1F**). Further studies revealed that eIF4B^-/-^ embryos died between E14.5 and E16.5, showing severe embryonic dysplasia, compared with eIF4B^+/-^ and WT embryos (**Figures 1G–I**). Subsequently, we examined E14.5 embryos of eIF4B^-/-^, and found that these embryos were pale and their size was smaller than those of WT ones (**Supplementary Figure 1C**), indicating that eIF4B KO inhibits the development of mouse embryos. Livers of eIF4B^-/-^ embryos were much smaller than those of the WT embryos, as indicated by much lower weight and dramatically decreased total cell number of the livers (**Supplementary Figures 1D, E**). Taken together, the results indicate that homozygous deletion of eIF4B (eIF4B^-/-^) is embryonic lethal, but heterozygous deletion of eIF4B (eIF4B^+/-^) is viable and eIF4B^+/-^ mice develop normally.

### Depletion of eIF4B Markedly Increases the Mortality of Adult Mice

**Figure 2:**
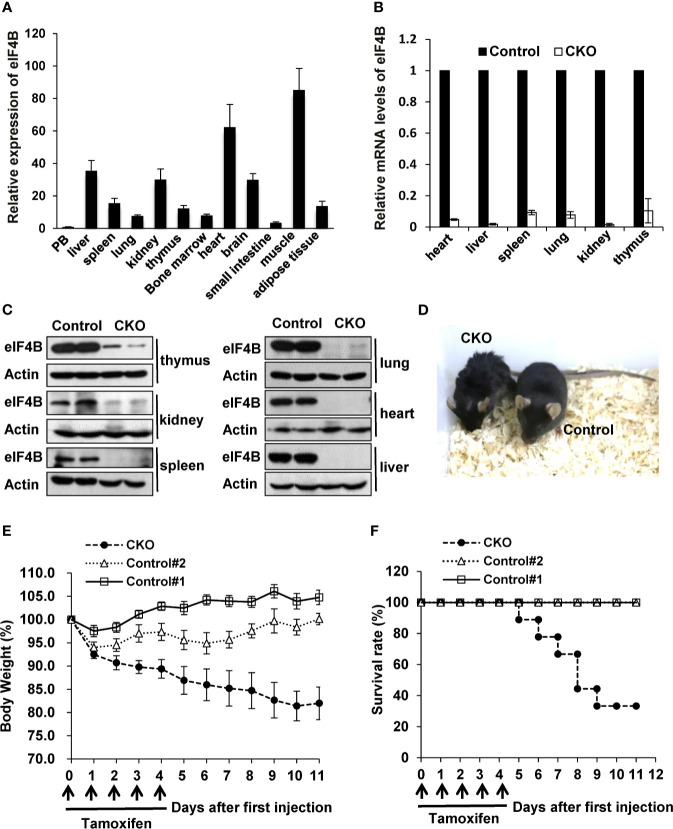
Depletion of eIF4B markedly increases the mortality of adult mice. **(A)** Relative expression of eIF4B in different organs, was detected by qRT-PCR (n = 3). Data are represented as mean ± SD. **(B, C)** mRNA and protein levels of eIF4B in indicated tissues of eIF4B CKO mice and control littermates, were detected by qRT-PCR **(B)** and Western blotting **(C)**. **(D)** Shown is the representative photo of eIF4B CKO mice and control littermates treated with tamoxifen. **(E, F)** Shown are the body weight change **(E)** and survival rate **(F)** of eIF4B CKO mice and control littermates (8-10 mice per group). “Control#1” represents “eIF4B^f/f^, Cre + Corn oil” group, “Control#2” represents “eIF4B^f/f^ + Tamoxifen” group, “CKO” represents “eIF4B^f/f^, Cre + Tamoxifen” group.

Since homozygous deletion of eIF4B (eIF4B^-/-^) in mice is embryonic lethal, we asked whether eIF4B deficiency had any effects on the development and physiological activity in adult mice. To this end, we crossed eIF4B^f/f^ mice with UBC-CreER^T2^ transgenic mice carrying a tamoxifen-inducible recombinase gene (**Supplementary Figures 2A, B**). Firstly, expression of eIF4B in multiple tissues was examined by qRT-PCR, and relatively high levels of eIF4B were found in several tissues such as muscle, heart and liver (**Figure 2A**). To deplete eIF4B in all mouse organs, 10-12 weeks-old eIF4B^f/f^ UBC-CreER^T2^ and eIF4B^f/f^ littermate mice were treated with tamoxifen for five consecutive days by intraperitoneal injection. After tamoxifen treatment, we examined eIF4B mRNA and protein levels in multiple organs from eIF4B^f/f^ UBC-CreER^T2^ (eIF4B CKO) and eIF4B^f/f^ littermate (control) mice. Overall, more than 95% reduction of eIF4B was observed in all organs of eIF4B CKO mice (**Figures 2B, C**). The eIF4B CKO mice showed abnormal phenotypic behaviors, characterized by matted hairs, shrinking and backend bow, loss of motility, and loss of appetite as compared with control mice (**Figure 2D**). Moreover, eIF4B CKO mice displayed more body weight loss than control groups after the first injection of tamoxifen (**Figure 2E**). The majority of eIF4B CKO mice died within 10 days, while control animals survived under the same condition (**Figure 2F**). The results indicate that depletion of eIF4B is able to lead to death of adult mice.

### eIF4B Deficiency Results in a Significant Decrease of Immune Cells

**Figure 3:**
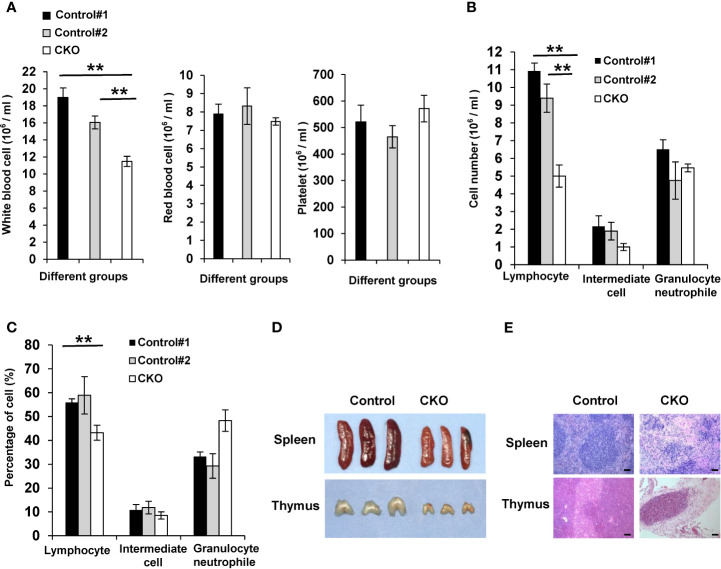
eIF4B deficiency results in a significant decrease of immune cells. **(A)** The number of blood cells from the peripheral blood of eIF4B CKO mice and control littermates was counted using an automatic blood cell analyzer (n = 7-10). Data are represented as mean ± SD. **p ≤ 0.01. **(B, C)** The number **(B)** and percentage **(C)** of different types of WBC (lymphocytes, intermediate cells and granulocyte neutrophils) of eIF4B CKO mice and control littermates were analyzed by complete blood cell counting (n = 7-10). Data are represented as mean ± SD. **p ≤ 0.01. **(D, E)** Representative images of spleen and thymus **(D)** and tissue sections **(E)** of eIF4B CKO mice and control littermates. Scale bars, 100 µm. “Control#1” represents “eIF4B^f/f^, Cre + Corn oil” group, “Control#2” represents “eIF4B^f/f^ + Tamoxifen” group, “CKO” represents “eIF4B^f/f^, Cre + Tamoxifen” group.

We next wondered whether the deficiency of eIF4B causes severe pathological damage in adult mice, leading to death of the animals. To this end, pathological analysis was performed for multiple organs. Unexpectedly, the gross examination and tissue structures of some organs including lung, liver and kidney appeared to be normal in eIF4B CKO mice, compared to control mice (**Supplementary Figures 3A, B**). To further determine how eIF4B deficiency results in high mortality in mice, we examined peripheral blood cells of eIF4B CKO mice and control littermates using an automatic blood cell analyzer. It turned out that eIF4B deficiency caused a significant decrease in the number of white blood cells (WBC) in eIF4B CKO mice, despite no significant effect on the numbers of red blood cells and platelets (**Figure 3A**). Also, both total number and the percentage of lymphocytes decreased significantly in peripheral blood cells of eIF4B CKO mice compared with control littermates (**Figures 3B, C**). In line with this, spleen and thymus were relatively smaller in eIF4B CKO mice than in control littermates (**Figure 3D**). In addition, we analyzed the changes in immune cell subsets (T cells and B cells) in the spleen and thymus by flow cytometry staining. The results showed that no significant difference was observed between control mice and eIF4B CKO mice (**Supplementary Figures 3C, D**). Pathological analysis showed a significant decrease in lymphocytes in spleen and thymus of eIF4B CKO mice (**Figure 3E**). These data suggest that atrophy of immune organs including spleen and thymus and the significant decline in the number of immune cells may contribute to the high mortality of eIF4B-deficient adult mice.

### eIF4B-Deficient Mice Are Highly Susceptible to Viral Infection

**Figure 4:**
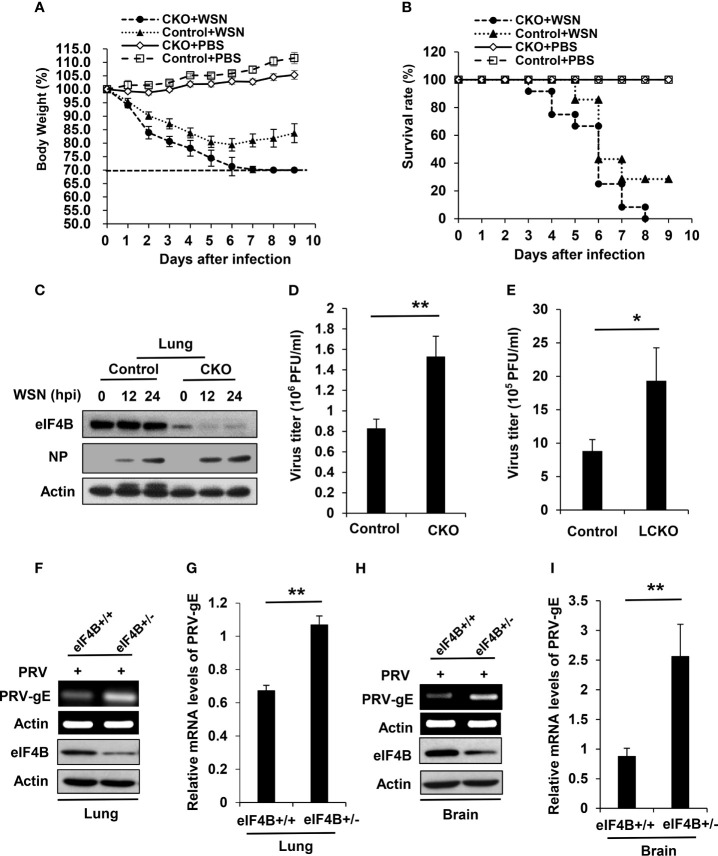
eIF4B-deficient mice are highly susceptible to viral infection. **(A, B)** Shown are body weight change **(A)** and survival rate **(B)** of eIF4B CKO and control mice intranasally infected with/without WSN. **(C)** Western blot analysis of eIF4B and IAV NP (Nucleoprotein) levels in lung lysates from eIF4B CKO and control mice infected with/without WSN virus. **(D)** Lung viral loads in infected eIF4B CKO and control mice were measured by plaque forming assay. **(E)** Viral loads in the lungs of eIF4B^f/f^ SPC-rtTA/Teto-Cre (eIF4B LCKO) and eIF4B^f/f^ (control) mice infected with WSN. **(F, G)** eIF4B^+/+^ and eIF4B^+/-^ mice were infected with PRV. The expression levels of PRV-gE in the lungs were analyzed by RT-PCR **(F)** and qRT-PCR **(G)**. **(H, I)** The expression levels of PRV-gE in the brains from eIF4B^+/+^ and eIF4B^+/-^ mice infected with PRV were analyzed by RT-PCR **(H)** and qRT-PCR **(I)**. Data are represented as mean ± SD. **p ≤ 0.01 and *p ≤ 0.05.

Our previous study has shown that altering eIF4B expression profoundly impacts influenza virus replication *in vitro* by regulating the expression of IFITM3 (40). To determine the effect of eIF4B on viral infection *in vivo*, eIF4B CKO and control mice were infected with equal amount of WSN virus. Under the same experimental condition, eIF4B CKO mice lost more body weight than the control mice (**Figure 4A**). In addition, all infected eIF4B CKO mice died within 8 days, whereas approximately 30% infected control mice still survived at the end of the experiments (**Figure 4B**). Supportively, the protein levels of viral NP in the lungs of eIF4B CKO mice were higher than those in control mice (**Figure 4C**). Consistent with these observations, the viral titers in the lungs increased significantly in eIF4B CKO mice compared to control mice (**Figures 4D** and **Supplementary Figure 4A**). On the other hand, we generated another mouse model in which eIF4B gene was conditionally disrupted in lung epithelium by crossing eIF4B^f/f^ mice with SPC-rtTA/Teto-Cre mice and then treating with doxycycline. Then eIF4B^f/f^ SPC-rtTA/Teto-Cre (named eIF4B LCKO) mice and eIF4B^f/f^ mice (control) treated with doxycycline were infected with WSN virus and examined for virus titers in the lungs. Deficiency of eIF4B in lung epithelium significantly increased IAV replication (**Figure 4E**). Additionally, eIF4B^+/+^ and eIF4B^+/-^ mice were infected with pseudorabies *virus* (PRV), and PRV replication was determined by analyzing the mRNA level of PRV-gE in mouse lungs and brains by RT-PCR and qRT-PCR. Similarly, the mRNA levels of PRV-gE in the lungs (**Figures 4F, G**) and brains (**Figures 4H, I**) from eIF4B^+/-^ mice significantly increased compared to those from eIF4B^+/+^ mice. These experiments demonstrate that eIF4B-deficient mice are more susceptible to viral infection.

### Knockout of eIF4B Enhances Pulmonary Inflammation Induced by Viral Infection

**Figure 5:**
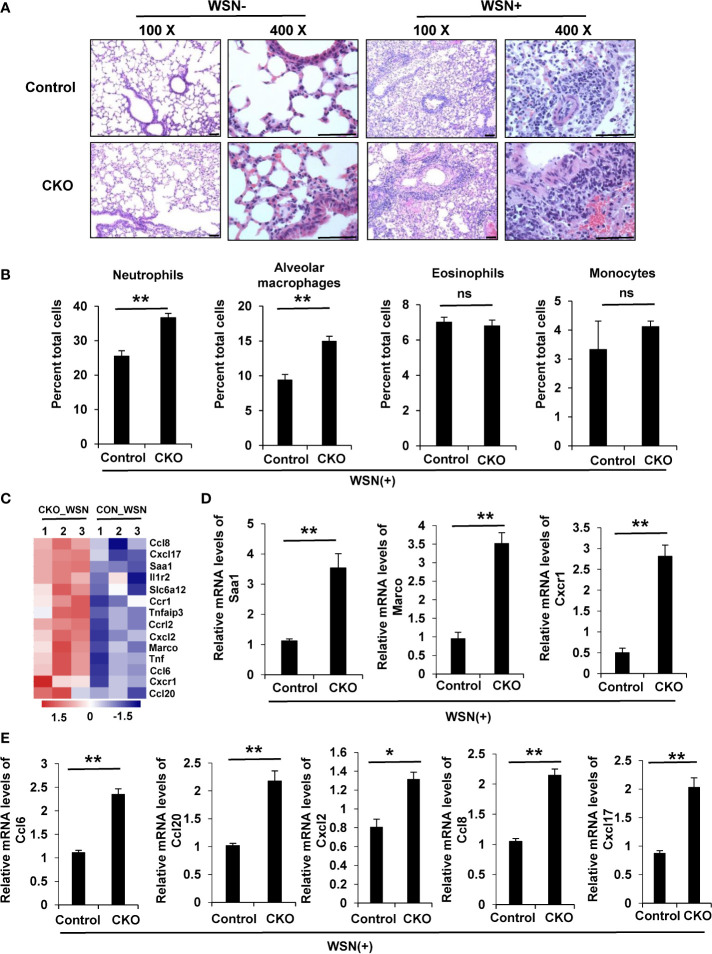
Knockout of eIF4B enhances pulmonary inflammation induced by virus infection. **(A)** Representative HE-staining images of lung tissue sections from eIF4B CKO and control mice with or without viral infection. Scale bars, 100 µm. **(B)** Flow cytometry of single-cell suspensions of the lungs from eIF4B CKO and control mice infected with WSN. **(C)** Shown are the differentially expressed inflammation-associated factors in WSN-infected lungs of eIF4B CKO mice compared to those in control mice. **(D, E)** Levels of several important inflammation-associated factors **(D)** or chemokines **(E)** were examined by qRT-PCR. Data are represented as mean ± SD. ns, not significant; **p ≤ 0.01 and *p ≤ 0.05.

Since eIF4B knockout mice were highly susceptible to viral infection (**Figure 4**), we next evaluated the effect of eIF4B on the pathogenesis of influenza A virus in the lungs. As predicted, eIF4B CKO mice exhibited a high degree of acute lung injury induced by influenza virus infection, compared to the WT animals (**Supplementary Figure 5A**). In line with this, histological analysis showed more severe inflammation and inflammatory cell infiltration in the lungs of eIF4B CKO mice (**Figure 5A**). Furthermore, flow cytometric analysis of single-cell suspensions isolated from the lungs of mice revealed that the number of neutrophils or alveolar macrophages significantly increased in the lungs of eIF4B CKO mice infected with WSN virus (**Figure 5B**). However, we observed that percentage of CD4+ and CD8+ lymphocytes was reduced in lungs of eIF4B CKO mice as compared with that in WT animals (**Supplementary Figure 5B**). Additionally, eIF4B CKO and control mice were inoculated intranasally with PRV and cell suspensions from the lungs were analyzed by flow cytometry. Similarly, the percentages of neutrophils, alveolar macrophages and monocytes increased in the lungs of infected eIF4B CKO mice (**Supplementary Figure 5C**). Overall, the results indicate that knockout of eIF4B markedly enhances pulmonary inflammation in response to viral infection.

To determine the underlying mechanism of enhanced pulmonary inflammation in eIF4B CKO mice during the viral infection, we performed transcriptomic analysis by RNA-seq of lung tissues of eIF4B CKO and WT control mice infected with WSN (**Supplementary Figures 5D, E**). A total of 1836 genes appeared to be differentially expressed (DEGs) comprising 971 upregulated and 865 downregulated genes (**Supplementary Figure 5F**) in the lungs of infected eIF4B KO, compared with the control. Interestingly, further analysis revealed that depletion of eIF4B markedly increased the expression of several important inflammation-associated factors, such as serum amyloid A1 (Saa1), Marco, and Cxcr1 (**Figure 5C**). This finding was also confirmed by qRT-PCR (**Figures 5D**). Besides, the levels of several critical chemokines involved in recruitment/activation of neutrophiles, monocytes or macrophages, including Ccl6, Ccl8, Ccl20, Cxcl2 and Cxcl17, were also significantly elevated in the lungs of IAV infected eIF4B CKO mice as compared to control animals through both transcriptome analysis (**Figure 5C**) and qRT-PCR (**Figure 5E**). Together, these data suggest that disruption of eIF4B may cause excessive production of inflammation-associated factors and chemokines during the viral infection, which might lead to severe pulmonary inflammation in the animals. Moreover, we employed the dextran sulfate sodium (DSS)-induced colitis model and sought to examine the role of eIF4B in colonic inflammation. Interestingly, monitoring the body weight daily over the course of DSS treatment (**Supplementary Figure 5G**), measuring the colon length at the indicated day (**Supplementary Figure 5H**), and HE staining of colon tissues from eIF4B CKO and control mice (**Supplementary Figures 5I**), revealed that eIF4B-deficient mice exhibited more severe DSS-induced colonic injury than control mice, as evidenced by more weight loss, shorter colon length and more severe inflammation in eIF4B CKO mice. These observations further suggest that eIF4B plays a key role in regulating tissue inflammation in mice.

Additionally, we also observed that expression of TNF alpha-induced protein 3 (Tnfaip3), also known as A20, was robustly increased in both lungs of eIF4B CKO mice and eIF4B^-/-^ cells after IAV infection compared with that in WT controls (**Figures 5C**, **Supplementary Figures 6A, B**). It has been shown that Tnfaip3 plays a key role in suppressing IAV-induced innate immune response and deficiency of Tnfaip3 in myeloid cells or lung epithelial cells protects against IAV infection in mice (50, 51). Therefore, we performed experiments using cell lines A549 and THP1, and observed that IAV infection remarkably upregulated the expression of Tnfaip3 (**Supplementary Figures 6C, D**). Furthermore, silencing endogenous Tnfaip3 in A549 cells with specific shRNAs significantly inhibited IAV replication (**Supplementary Figures 6E–G**). These observations suggest that eIF4B is involved in regulating Tnfaip3 expression, and the elevated level of Tnfaip3 in IAV infected eIF4B-deficient mice is associated with the increased susceptibility of the animals to viral infection.

### Deficiency of eIF4B Impairs NK Cell Mediated Cytotoxicity in Mice

**Figure 6:**
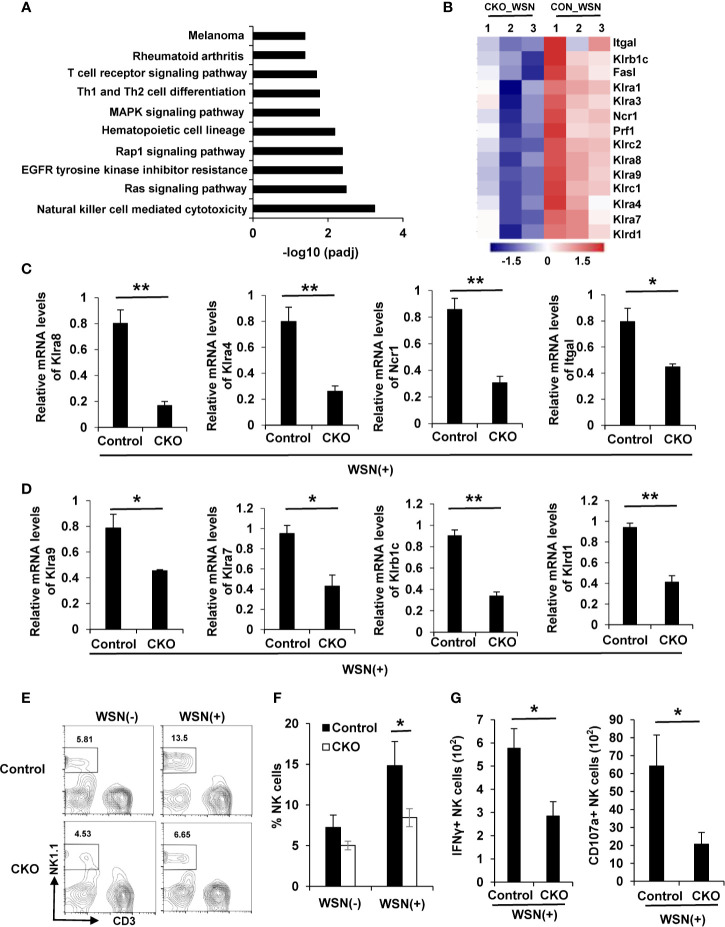
Deficiency of eIF4B impairs NK cell mediated cytotoxicity in mice. **(A)** Potential pathways for differentially expressed genes (DEGs) from transcriptome RNA sequencing, were examined by KEGG enrichment analysis. **(B)** Shown are the differentially expressed activating or inhibitory receptors of NK cells in WSN-infected lungs of eIF4B CKO mice as compared to control mice. **(C, D)** mRNA levels of activating **(C)** or inhibitory **(D)** receptors were detected by qRT-PCR. Shown are three independent experiments. **(E, F)** The percentage of NK cells in the lungs of uninfected or infected eIF4B CKO mice and control mice, was analyzed by flow cytometry. **(G)** The number of IFN-γ+ and CD107a+ NK cells in total NK cells. Data are represented as mean ± SD. **p ≤ 0.01 and *p ≤ 0.05.

Since transcriptome RNA-seq identified 1836 DEGs in the lungs of eIF4B CKO mice in response to IAV infection, potential pathways for these DEGs were further explored by KEGG enrichment analysis. Interestingly, natural killer (NK) cell mediated cytotoxicity pathway was the most significantly enriched in these samples (**Figure 6A**). NK cells are lymphoid effectors of innate immune system and shown to have the specific ability to rapidly kill virus-infected host cells (52), which is implicated in control of virus pathogenesis (53, 54). Specially, NK cells express a variety of activating or inhibitory receptors (55). Here, transcriptome analysis revealed that several crucial receptors expressed in NK cells, including activating and inhibitory receptors, were markedly downregulated in the lungs of eIF4B CKO mice in comparison with control mice (**Figure 6B**). This observation was further confirmed by real time PCR (**Figures 6C, D**). Next, we determined the infiltration of NK cells in the lungs of IAV-infected eIF4B CKO and control mice. IAV infection increased the percentage of NK cells in the lungs of both the control and eIF4B CKO mice, but CKO significantly attenuated IAV-induced NK cells in the lungs (**Figures 6E, F**). Additionally, we further determined NK cell activation in the lungs of control and eIF4B CKO mice by analysis of IFN-γ and CD107a expression level. The results showed that the number of IFN-γ+ and CD107a+ NK cells in eIF4B CKO mice was significantly decreased compared to that in control mice (**Figures 6G**, **Supplementary Figure 6H**). Taken together, these observations indicate that knockout of eIF4B impairs the infiltration of NK cells and their function in the lung in response to IAV infection, which may result in high susceptibility of the eIF4B CKO animals to the viral infection.

## Discussion

Translational regulation plays a vital role in modulating cell growth and survival, as well as the interaction of virus and host cells (2, 4, 56). It is known that translation initiation acts as the rate-limiting step of protein synthesis and translation initiation factors are often targeted by multiple regulatory mechanisms (3). Extensive molecular and cellular studies have demonstrated that eIF4B is an intricate eukaryotic translation initiation factor, which controls translation of some specific mRNAs with complex secondary structures and is involved in various cellular events (6, 26, 57). However, the role of eIF4B at the organismal level is still unknown. In this study, we, for the first time, present evidence that mice with heterozygous deletion of eIF4B (eIF4B^+/-^) were viable and phenotypically similar to WT (eIF4B^+/+^) littermates. However, homozygous deletion of eIF4B (eIF4B^-/-^) led to mouse embryonic lethality and eIF4B^-/-^ embryos died between E14.5 and E16.5. The finding indicates that eIF4B is required for embryonic development in mice. This is in agreement with previous studies showing that translation control not only plays a critical role in regulating tumor cell survival and proliferation, but also is required for normal development and physiology *in vivo* (58, 59). It has also been described that complete loss of eIF4E, a 5’ cap-binding protein that recruits eIF4G and eIF4A to assemble the eIF4F complex, is mouse embryonic lethal and eIF4E^-/-^ embryos die before E6.5 (59).

Fetal liver is an fundamentally hematopoietic organ and functions as a major site for hematopoietic stem cell (HSC) expansion during embryogenesis (60). Recent studies have shown that defect of fetal liver generally exhibits impaired HSC expansion and defective erythropoiesis and subsequently causes embryonic death or perinatal death (44, 61, 62). Here, we noticed that eIF4B^-/-^ embryos showed pale and small-sized fetal liver, accompanied by decreased fetal liver weight and cell number. Likely, abnormal development in fetal liver may be a major cause for the death of eIF4B^-/-^ embryos. Phosphorylation of eIF4B is regulated by several proto-oncogenic signaling molecules and subsequently affects cell growth and survival by modulating the translation of certain mRNAs, such as *CDC25C, c-myc, BCL2* and *XIAP* (21, 22, 26, 29, 38). Hence, further research is needed to understand whether eIF4B deficiency promotes cell apoptosis in fetal liver through regulating the expression of apoptosis-related proteins, leading to severe defect in fetal liver.

On the other hand, we found that conditional knockout (CKO) of eIF4B in adulthood profoundly increased the mortality of adult mice. Apparently, eIF4B CKO resulted in severe pathological damage in spleen and thymus and remarkably reduced the number of lymphocytes in peripheral blood. These results support the notion that eIF4B is required for the survival and development of adult mice in addition to embryonic development. However, it remains to be determined whether eIF4B CKO-induced reduction of immune cells is attributed to decreased cell proliferation and/or increased cell apoptosis.

The process of viral replication in infected cells relies on the translation machinery of the host. Therefore, translation control in host cells is crucial for viral infection and pathogenesis (56). To ensure the synthesis of viral proteins and production of progeny virion, viruses have evolved diverse strategies to subvert host translation machinery, so targeting translation initiation factors has become one of the most effective strategies to fight against viral infection (63–66). Innate immunity provides the first line of defense against viral infection (47). Our previous data have suggested that eIF4B is involved in antiviral innate immunity through regulating the translation of *IFITM3* (40). In this study, we demonstrated that eIF4B CKO mice were more susceptible to viral infection including influenza virus and pseudorabies virus. Importantly, the specific depletion of eIF4B in lung epithelium (eIF4B LCKO) also significantly enhanced IAV replication in the lung. Furthermore, we found that eIF4B deficiency resulted in a significantly increased expression of inflammation-associated factors Saa1, Marco and Cxcr1, and chemokines Ccl6, Ccl8, Ccl20, Cxcl2 and Cxcl17. These results are in accordance with the severe inflammatory cell (neutrophils and macrophages) infiltration in the lungs of eIF4B CKO mice. Saa1 is an acute phase protein playing a key role in many inflammatory diseases including those caused by IAV and SARS-CoV-2 infection (67). Previous studies reported that Saa1 could activate and recruit neutrophils to the lung in infectious diseases (68). Marco is a scavenger receptor protein expressing in naïve tissue-resident macrophages, including lung alveolar macrophages (69), and Cxcr1 is an important chemokine receptor involved in the recruitment of neutrophils (70). NK cells play important roles in mediating host innate immune response to virus infection (71). Moreover, several studies have highlighted that decrease of NK cells or defects in NK cell activity result in higher morbidity and mortality, owing to inefficient viral clearance (54, 72). Surprisingly, we observed that eIF4B knockout impaired NK cells infiltration and their function in the lung infected with IAV. Additionally, transcriptomic analysis by RNA-seq showed that knockout of eIF4B affected expression of genes related to T cell signaling and Th differentiation. The differentiation of specialized effector Th cells not only plays an important role in regulating the development of effector and memory CD8+ T cells and B cell response, but also is involved in antiviral immune responses against various pathogens (73). However, the mechanism underlying T cell defect in eIF4B CKO mice still needs to be further investigated. Taken together, these data suggest that eIF4B is required for antiviral immunity likely through multiple mechanisms.

In summary, we have for the first time determined the function of eIF4B at the organismal level by generation of eIF4B germline knockout and conditional knockout mouse models. Our results reveal that eIF4B is critically required for mouse embryonic development, as indicated by the results that homozygous deletion of eIF4B leads to mouse embryonic death related to defective development of fetal liver. In addition, eIF4B is also essential for the animal survival, and antiviral responses of adult mice, as evidenced by markedly increased mortality of eIF4B conditional knockout adult mice, and significantly enhanced susceptibility of eIF4B-deficient animals to viral infection. Our findings establish critical roles for eIF4B in the regulation of normal development and antiviral immunity.

## Data Availability Statement

The datasets presented in this study can be found in online repositories. The names of the repository/repositories and accession number(s) can be found below: https://www.ncbi.nlm.nih.gov/, GSE166944.

## Ethics Statement

The animal study was reviewed and approved by Research Ethics Committee of Institute of Microbiology, Chinese Academy of Sciences.

## Author Contributions

BC designed and performed experiments, analyzed the data, and wrote the manuscript. YC, KR, and XW helped in data analysis and revised the manuscript. SL, YL, MX, YM, and GW participated in some experiments. GG and SH revised the manuscript. J-LC designed and supervised the project, wrote and revised the manuscript. All authors contributed to the article and approved the submitted version.

## Funding

This work was supported by National Science Foundation of China (U1805231, 32030110) and Science and Technology Innovation Project of FAFU (CXZX2018014).

## Conflict of Interest

The authors declare that the research was conducted in the absence of any commercial or financial relationships that could be construed as a potential conflict of interest.

## Publisher’s Note

All claims expressed in this article are solely those of the authors and do not necessarily represent those of their affiliated organizations, or those of the publisher, the editors and the reviewers. Any product that may be evaluated in this article, or claim that may be made by its manufacturer, is not guaranteed or endorsed by the publisher.

## Supplementary Material

Supplementary Figure 1 Knockout of eIF4B leads to mouse embryonic lethality. **(A)** Western blotting analysis of eIF4B protein levels in the indicated tissues (kidney, thymus and spleen) of eIF4B^+/-^ mice and their eIF4B^+/+^ littermates. **(B)** Representative micrographs of the indicated organs (kidney, thymus and spleen) from eIF4B^+/-^ mice and their eIF4B^+/+^ littermates, which were stained with hematoxylin and eosin (HE). **(C)** Representative images of E14.5 embryos and fetal liver. **(D)** Shown are absolute weight **(D)** and cell number **(E)** of fetal liver from eIF4B^-/-^ embryos and eIF4B^+/+^ littermates (n = 3-5 per genotype for each group). Data are represented as mean ± SD. ***p ≤ 0.001 and **p ≤ 0.01.

Supplementary Figure 2 Depletion of eIF4B markedly increases the mortality of adult mice. **(A)** Schematic representation to obtain eIF4B conditional knockout mice by breeding eIF4B^f/f^ mice with UBC-CreER^T2^ mice. **(B)** Genotyping of eIF4B^f/f^ mice.

Supplementary Figure 3 eIF4B deficiency results in a significant decrease of immune cells. **(A)** Representative images of kidney, liver and lung of eIF4B CKO mice and control littermates. **(B)** Representative images of HE staining of kidney, liver and lung of eIF4B CKO mice and control littermates. **(C, D)** Single-cell suspensions were obtained from spleens and thymuses of eIF4B CKO and control mice, and the percentage of CD4+ T cells, CD8+ T cells and CD19+ B cells were analyzed by flow cytometry.

Supplementary Figure 4 eIF4B-deficient mice are highly susceptible to viral infection. **(A)** Lung viral loads in infected eIF4B^+/-^ mice and their eIF4B^+/+^ littermates were determined by plaque forming assay. Data are represented as mean ± SD. **p ≤ 0.01 and *p ≤ 0.05. **(B)** Western blotting analysis of eIF4B protein levels in the indicated tissues (heart, thymus, spleen and liver) of control and eIF4B CKO mice. Tamoxifen was injected at a dose of 100 μg/g body weight for three days.

Supplementary Figure 5 Knockout of eIF4B enhances pulmonary inflammation induced by virus infection and DSS-induced colitis. **(A)** Representative gross images of lungs from eIF4B CKO and control mice with or without WSN virus infection. **(B, C)** Single-cell suspensions were obtained from the lungs of eIF4B CKO and control mice infected with WSN, and analyzed by flow cytometry. Data are represented as mean ± SD. **p ≤ 0.01 and *p ≤ 0.05. **(D)** Levels of eIF4B mRNA in lungs of WSN-infected eIF4B CKO mice and control littermates, were detected by qRT-PCR. **(E)** The cluster analysis of transcriptome RNA sequencing in the lungs of uninfected or infected eIF4B CKO mice and control mice, was performed. **(F)** Transcriptome RNA sequencing analysis identified 971 upregulated and 865 downregulated genes in WSN-infected lungs of eIF4B CKO mice compared to control mice. **(G)** Body weight was monitored daily during DSS-treatment period. **(H)** Colon length was measured at day 7. **(I)** Histopathological analysis was performed using colon samples. Data are represented as mean ± SD. **p ≤ 0.01 and *p ≤ 0.05.

Supplementary Figure 6 Tnfaip3 promotes IAV replication *in vitro*.**(A, B)** Relative mRNA levels of Tnfaip3 in WSN-infected lungs of eIF4B CKO and control mice **(A)** or in WSN-infected eIF4B^-/-^ and eIF4B^+/+^ MEFs **(B)**, were detected by qRT-PCR. **(C, D)** The expression levels of Tnfaip3 mRNA in A549 cells **(C)** or THP1 cells **(D)** challenged with WSN at indicated hours post-infection, were examined by qRT-PCR. **(E, F)** Tnfaip3 levels in A549 cells stably expressing pSIH-H1-GFP targeting Tnfaip3 or luciferase control, were examined by RT-PCR **(E)** or qRT-PCR **(F)**. **(G)** Culture supernatants from Tnfaip3 knockdown and control A549 cells, were harvested at the indicated hours post-infection and analyzed by haemagglutinin assay. **(H)** The percentage of IFN-γ+ and CD107a+ NK cells in total NK cells. Data are represented as mean ± SD. ns, not significant; **p ≤ 0.01 and *p ≤ 0.05.
