# Supplementary figures and images for: Deficiency of eIF4B Increases Mouse Mortality and Impairs Antiviral Immunity
Source: Front Immunol. 2021 Sep 10;12:723885. doi: 10.3389/fimmu.2021.723885 (PMC8461113; doi:10.3389/fimmu.2021.723885)

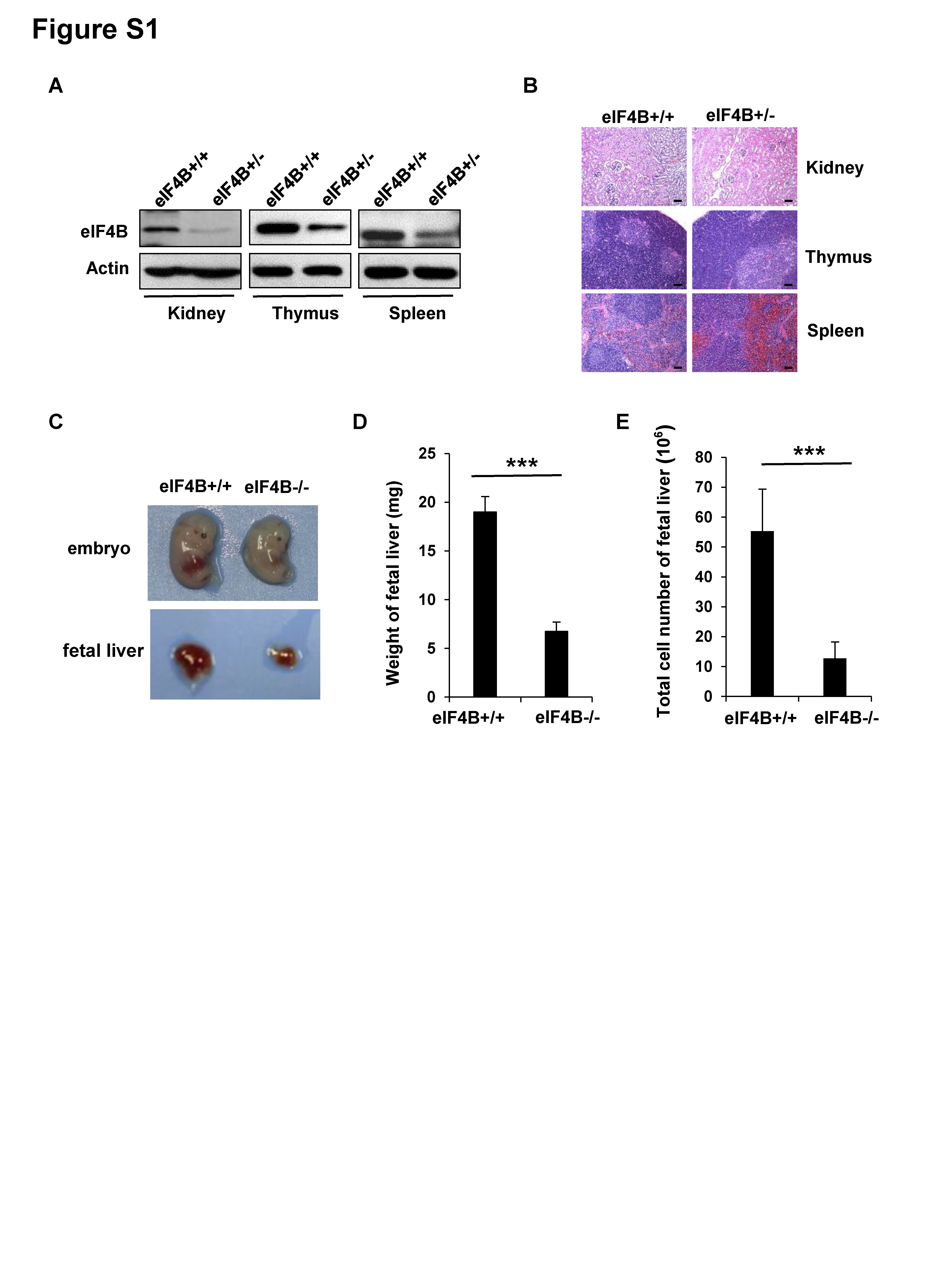

Supplement: Supplementary Figure 1 — Knockout of eIF4B leads to mouse embryonic lethality. (A) Western blotting analysis of eIF4B protein levels in the indicated tissues (kidney, thymus and spleen) of eIF4B+/- mice and their eIF4B+/+ littermates. (B) Representative micrographs of the indicated organs (kidney, thymus and spleen) from eIF4B+/- mice and their eIF4B+/+ littermates, which were stained with hematoxylin and eosin (HE). (C) Representative images of E14.5 embryos and fetal liver. (D) Shown are absolute weight (D) and cell number (E) of fetal liver from eIF4B-/- embryos and eIF4B+/+ littermates (n = 3-5 per genotype for each group). Data are represented as mean ± SD. ***p ≤ 0.001 and **p ≤ 0.01. [file Image_1.tif]

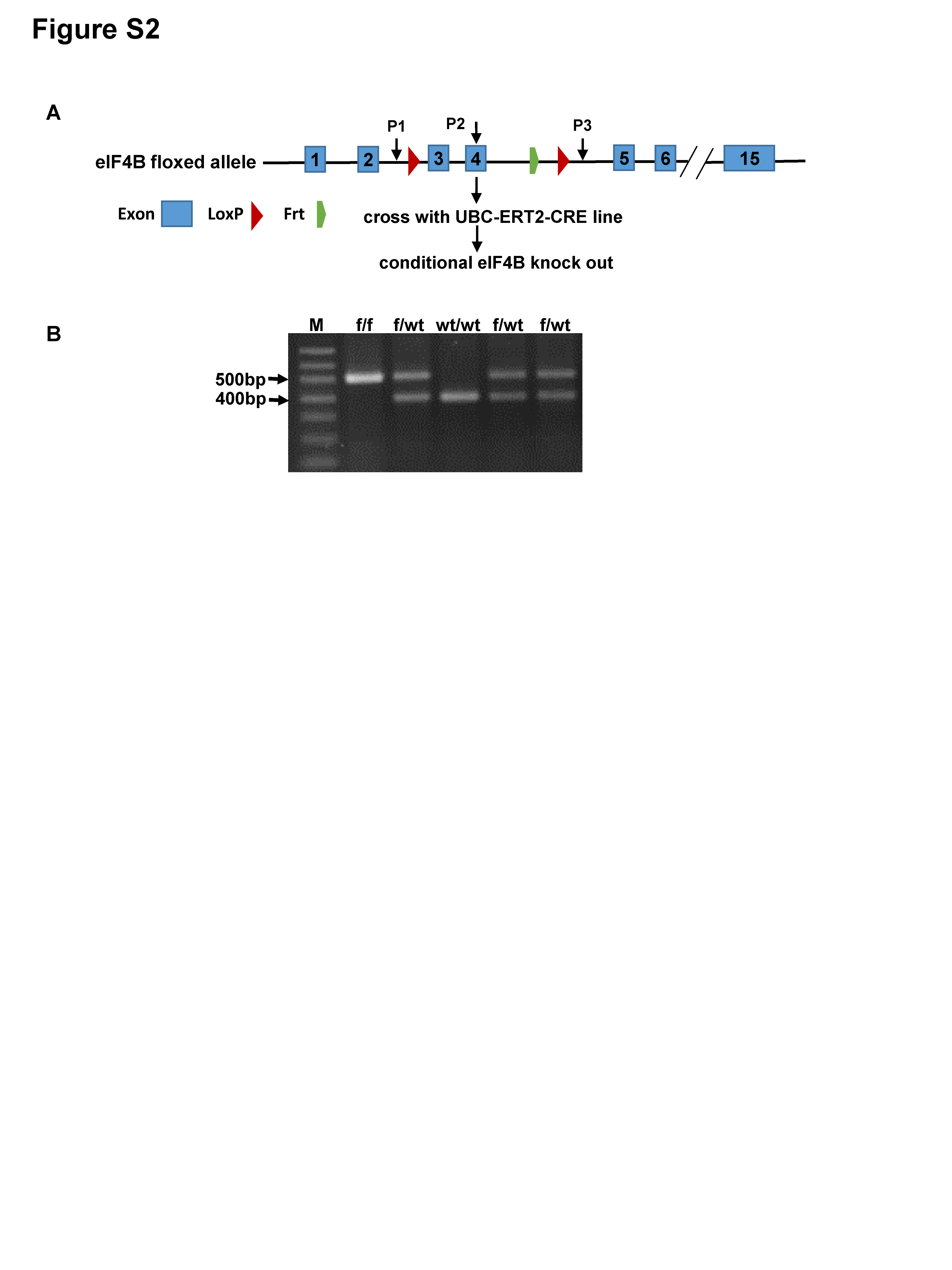

Supplement: Supplementary Figure 2 — Depletion of eIF4B markedly increases the mortality of adult mice. (A) Schematic representation to obtain eIF4B conditional knockout mice by breeding eIF4Bf/f mice with UBC-CreERT2 mice. (B) Genotyping of eIF4Bf/f mice. [file Image_2.tif]

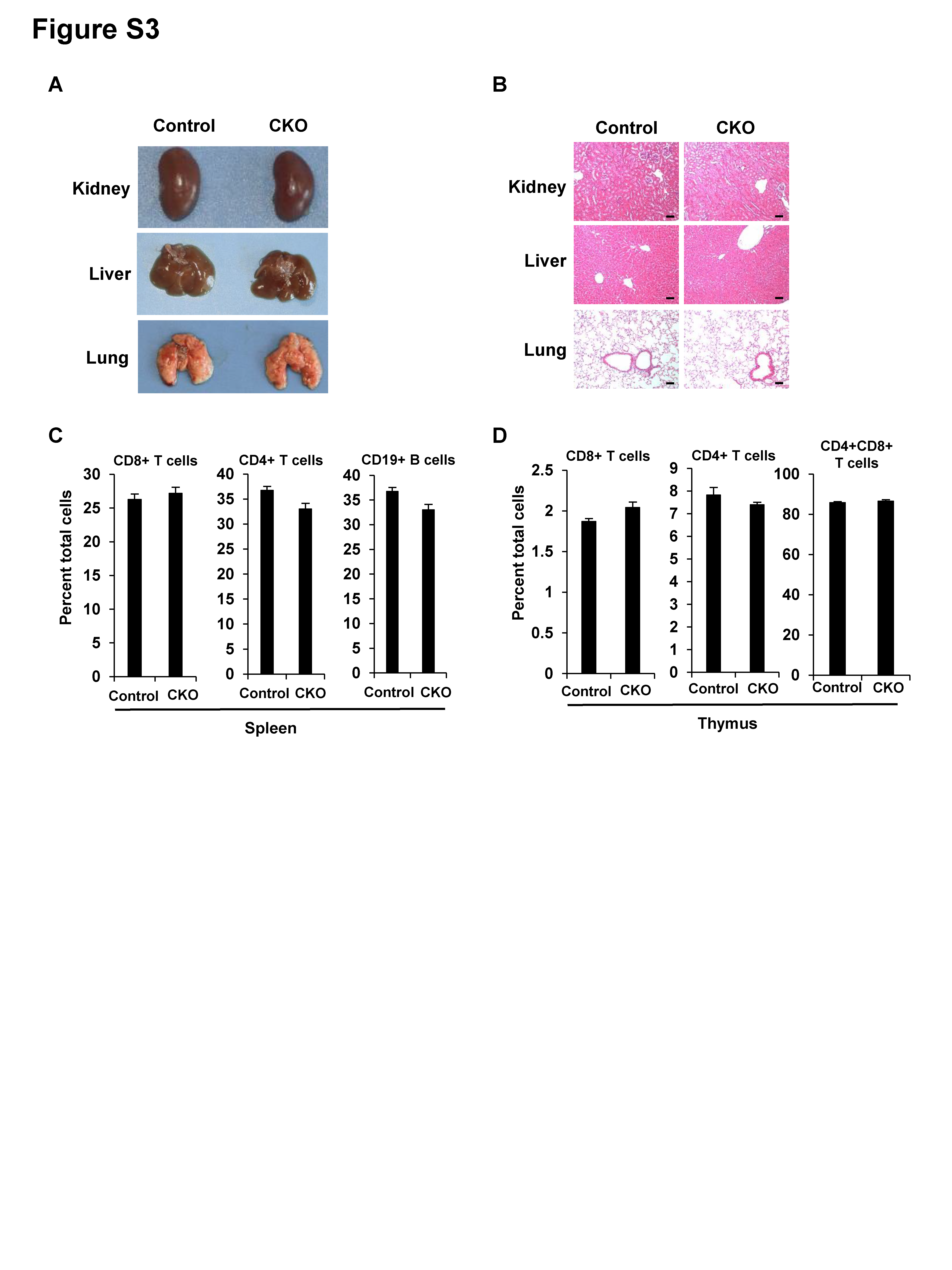

Supplement: Supplementary Figure 3 — eIF4B deficiency results in a significant decrease of immune cells. (A) Representative images of kidney, liver and lung of eIF4B CKO mice and control littermates. (B) Representative images of HE staining of kidney, liver and lung of eIF4B CKO mice and control littermates. (C, D) Single-cell suspensions were obtained from spleens and thymuses of eIF4B CKO and control mice, and the percentage of CD4+ T cells, CD8+ T cells and CD19+ B cells were analyzed by flow cytometry. [file Image_3.tif]

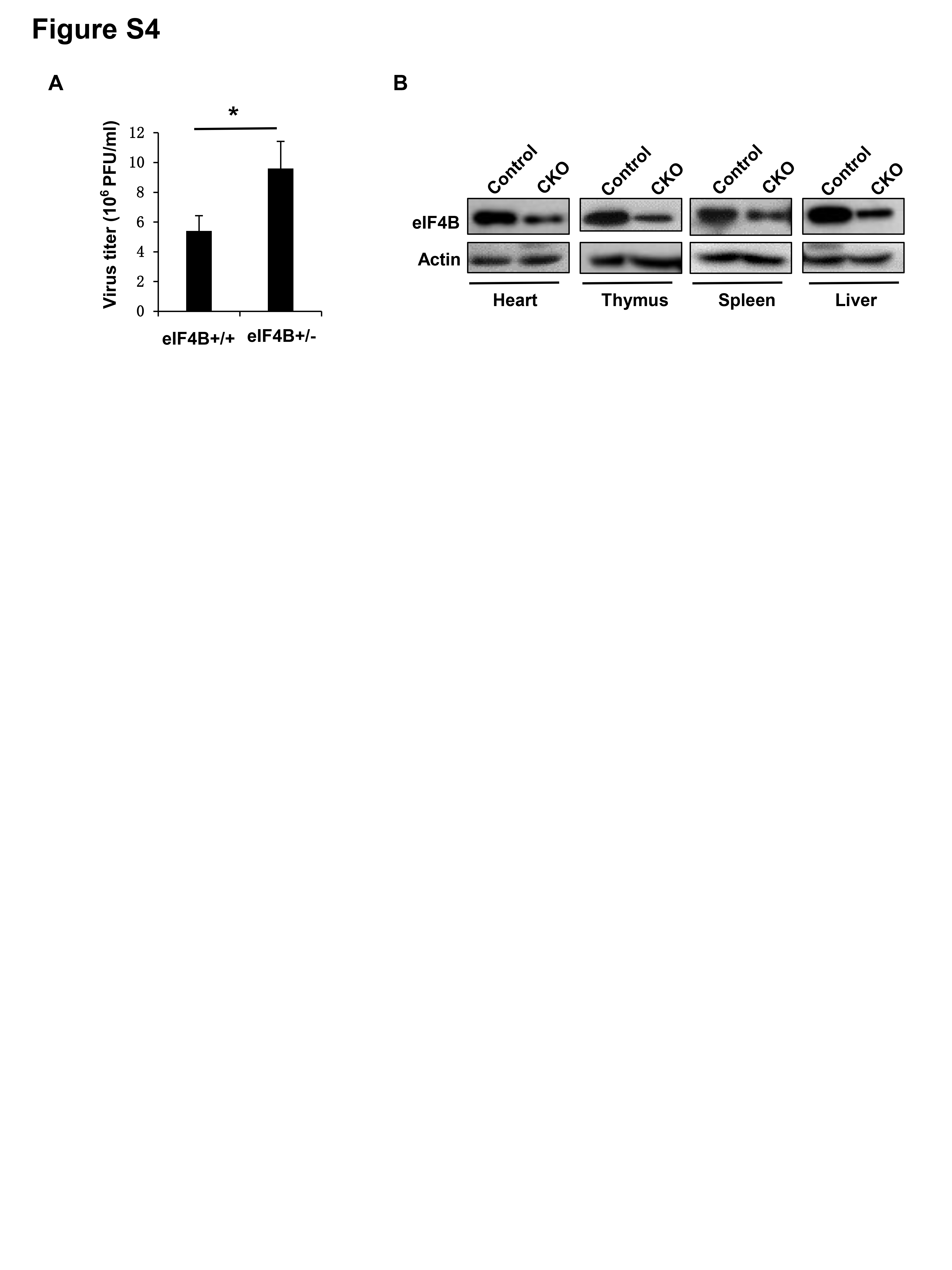

Supplement: Supplementary Figure 4 — eIF4B-deficient mice are highly susceptible to viral infection. (A) Lung viral loads in infected eIF4B+/- mice and their eIF4B+/+ littermates were determined by plaque forming assay. Data are represented as mean ± SD. **p ≤ 0.01 and *p ≤ 0.05. (B) Western blotting analysis of eIF4B protein levels in the indicated tissues (heart, thymus, spleen and liver) of control and eIF4B CKO mice. Tamoxifen was injected at a dose of 100 μg/g body weight for three days. [file Image_4.tif]

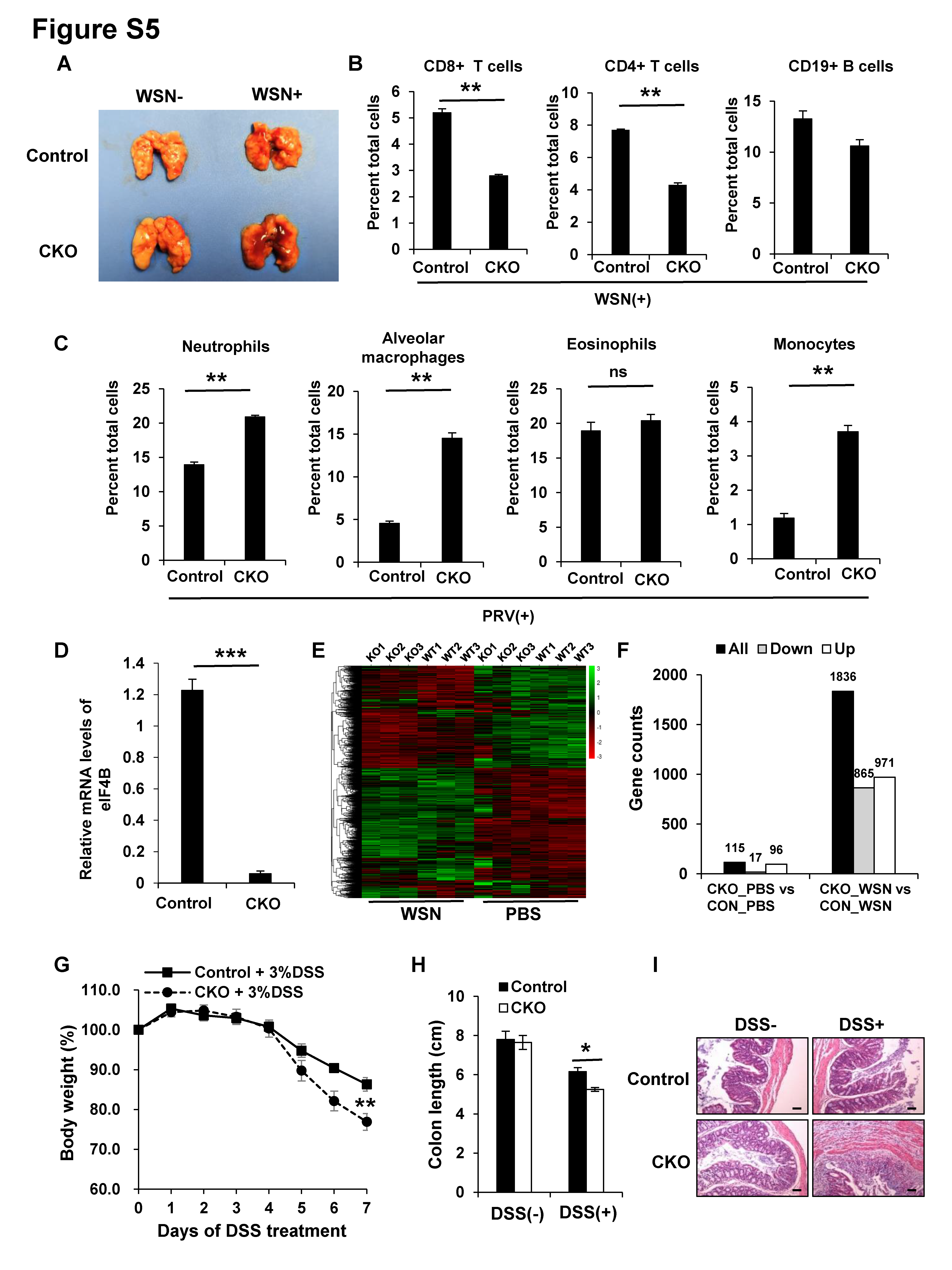

Supplement: Supplementary Figure 5 — Knockout of eIF4B enhances pulmonary inflammation induced by virus infection and DSS-induced colitis. (A) Representative gross images of lungs from eIF4B CKO and control mice with or without WSN virus infection. (B, C) Single-cell suspensions were obtained from the lungs of eIF4B CKO and control mice infected with WSN, and analyzed by flow cytometry. Data are represented as mean ± SD. **p ≤ 0.01 and *p ≤ 0.05. (D) Levels of eIF4B mRNA in lungs of WSN-infected eIF4B CKO mice and control littermates, were detected by qRT-PCR. (E) The cluster analysis of transcriptome RNA sequencing in the lungs of uninfected or infected eIF4B CKO mice and control mice, was performed. (F) Transcriptome RNA sequencing analysis identified 971 upregulated and 865 downregulated genes in WSN-infected lungs of eIF4B CKO mice compared to control mice. (G) Body weight was monitored daily during DSS-treatment period. (H) Colon length was measured at day 7. (I) Histopathological analysis was performed using colon samples. Data are represented as mean ± SD. **p ≤ 0.01 and *p ≤ 0.05. [file Image_5.tif]

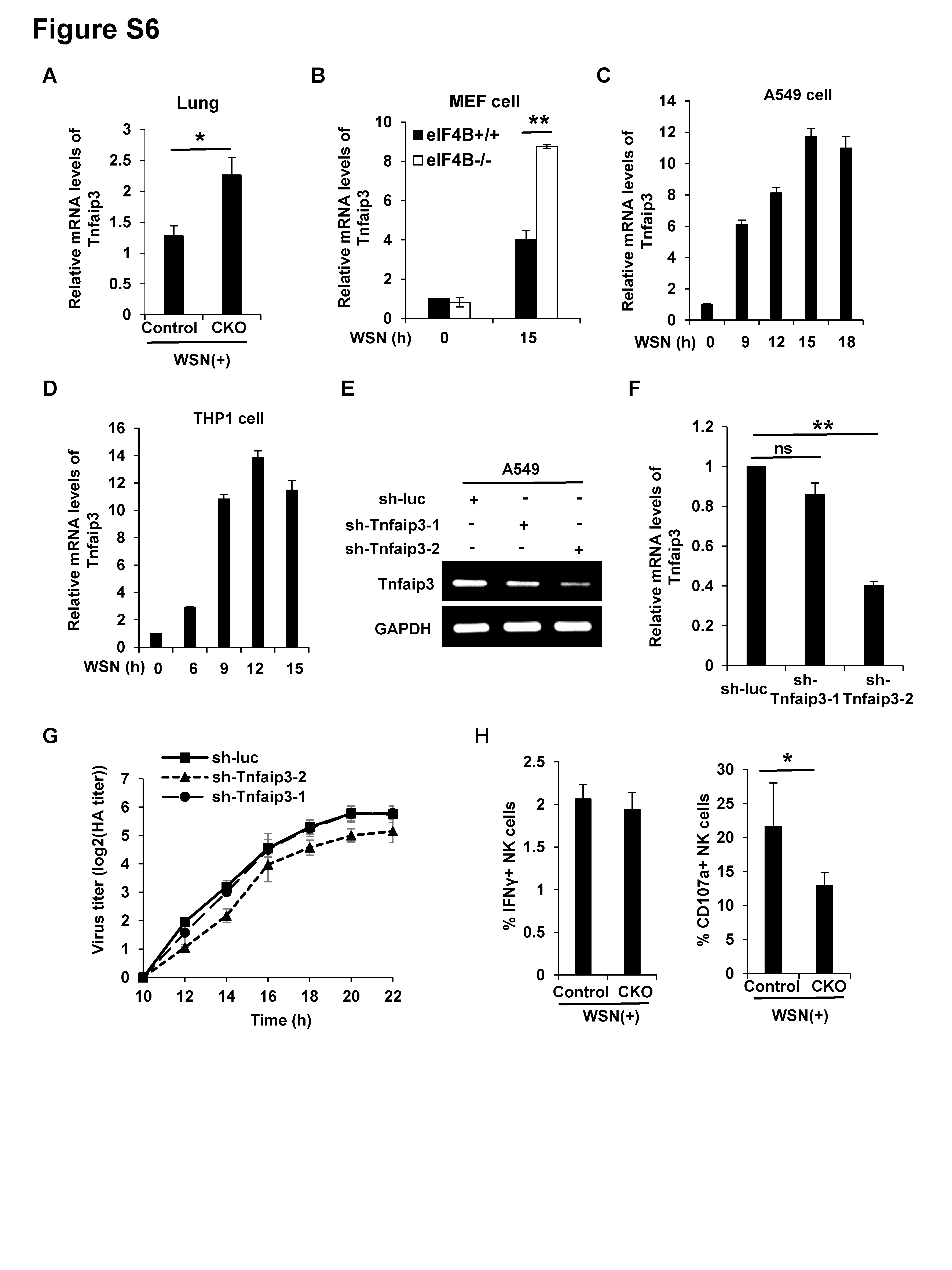

Supplement: Supplementary Figure 6 — Tnfaip3 promotes IAV replication in vitro.(A, B) Relative mRNA levels of Tnfaip3 in WSN-infected lungs of eIF4B CKO and control mice (A) or in WSN-infected eIF4B-/- and eIF4B+/+ MEFs (B), were detected by qRT-PCR. (C, D) The expression levels of Tnfaip3 mRNA in A549 cells (C) or THP1 cells (D) challenged with WSN at indicated hours post-infection, were examined by qRT-PCR. (E, F) Tnfaip3 levels in A549 cells stably expressing pSIH-H1-GFP targeting Tnfaip3 or luciferase control, were examined by RT-PCR (E) or qRT-PCR (F). (G) Culture supernatants from Tnfaip3 knockdown and control A549 cells, were harvested at the indicated hours post-infection and analyzed by haemagglutinin assay. (H) The percentage of IFN-γ+ and CD107a+ NK cells in total NK cells. Data are represented as mean ± SD. ns, not significant; **p ≤ 0.01 and *p ≤ 0.05. [file Image_6.tif]
